# Tobacco control in Türkiye: A brief review of achievements, challenges, and prospects

**DOI:** 10.18332/tpc/191783

**Published:** 2025-01-17

**Authors:** Ömer Faruk Sönmez

**Affiliations:** 1 School of Medicine and Population Health, University of Sheffield, Sheffield, United Kingdom

**Keywords:** tobacco control, Türkiye, tobacco industry, MPOWER, smoking prevalence, challenges

## Abstract

**INTRODUCTION:**

The global burden of tobacco is a significant public health concern, causing millions of deaths, illnesses, and economic losses annually. In Türkiye, tobacco use is deeply ingrained in society, with historical roots dating back to Ottoman times. The nation faces challenges such as high smoking rates, gender disparities, and the popularity of non-cigarette tobacco products. Despite these issues, Türkiye has adhered to the WHO Framework Convention on Tobacco Control (FCTC) and implemented MPOWER strategies to combat tobacco use effectively. This review aims to synthesize Türkiye's comprehensive tobacco control measures, including legislation, smoking cessation initiatives, public awareness campaigns, and taxation policies.

**METHODS:**

The study examines the country's strategic actions against the backdrop of its historical context and current challenges. The review leverages data from various sources, including the Global Burden of Disease, national health surveys, and reports on tobacco control efforts and achievements. Stakeholder activities, such as those by the Turkish Ministry of Health and non-governmental organizations, are also explored.

**RESULTS:**

Türkiye has made substantial progress in tobacco control, evidenced by reduced tobacco consumption and addressing key challenges like gender disparities and youth smoking. However, the country continues to face obstacles such as tobacco smuggling, the rise of narghile (hookah) smoking, and ongoing high daily smoking rates. The review highlights the tobacco industry’s influence in Türkiye despite strong adherence to FCTC.

**CONCLUSIONS:**

Türkiye's efforts in tobacco control represent a significant endeavor to mitigate the public health crisis posed by tobacco use. Through adherence to WHO FCTC guidelines and MPOWER strategies, notable progress has been achieved. Yet, challenges persist, requiring continuous, comprehensive strategies and robust enforcement. Future efforts must focus on strengthening tobacco control measures, ensuring accessibility to smoking cessation services, and countering the tobacco industry's influence, to further reduce tobacco use and its health and economic burdens.

## INTRODUCTION

Globally, the burden of tobacco products is a critical public health issue, affecting millions worldwide and leading to significant death, illness, and economic loss^1^. Tobacco, a leading cause of death and impoverishment, claims the lives of more than 8 million people annually, including approximately 1.3 million non-smokers exposed to secondhand smoke^2^. The epidemic is particularly severe in low- and middle-income countries, where around 80% of the world’s 1.3 billion tobacco users reside^3^. These countries bear the heaviest burden of tobacco-related illnesses and deaths^4^.

The use of tobacco, in any form, is universally harmful, with no safe level of exposure^5^. Cigarette smoking remains the most prevalent form of tobacco consumption, but other products like waterpipe tobacco, cigars, heated tobacco, and smokeless tobacco also contribute to the epidemic. Tobacco use not only leads to significant healthcare costs for treating diseases caused by tobacco^6^, but also impacts economic stability by diverting household spending from essential needs to tobacco, exacerbating poverty^7^.

The World Health Organization (WHO) has recognized the severity of the tobacco epidemic, and, in response, the WHO Member States adopted the WHO Framework Convention on Tobacco Control (WHO FCTC) in 2003, with 183 countries currently party to the treaty by June 2024^8^. Additionally, the WHO’s MPOWER measures align with the WHO FCTC, offering strategies that have been proven to save lives and reduce healthcare expenditure through averted tobacco use. Despite these efforts, the challenge of tobacco control remains a significant obstacle in safeguarding global public health.

**Figure 1:**
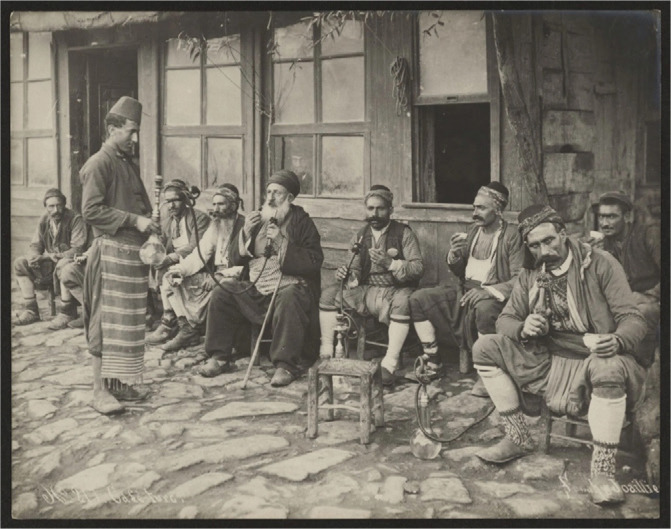
A traditional coffeehouse, men smoking narghile in Istanbul, Ottoman Empire, 1885^10^

In Türkiye, smoking is a socially ingrained behavior, especially among men, with its roots dating back to Ottoman times. The phrase ‘Smoking like a Turk’ used to be a popular expression across various European languages in the previous century. For Turks, however, tobacco has represented more than just a consumable product. It has served as a major source of revenue for over a century and has consistently attracted the interest of foreign investors due to its lucrative market potential. Traditionally, men would gather in coffeehouses to engage in conversation, play table games, and smoke (Figure 1), initially using hand-rolled cigarettes and waterpipes (narghile). The renowned Turkish folk poet Neşet Ertaş offers a culturally accepted perspective, defending smoking as a ‘stress relief method’ and a ‘simple pleasure for the poor’^9^. Tobacco control efforts in Türkiye had to focus on changing this narrative.

Even with extensive tobacco control measures in place, Türkiye remains the third highest consumer of tobacco among OECD countries^11^. Epidemiological data highlight the fluctuating nature of tobacco use in Türkiye and underscore the challenges in achieving a consistent downward trend in smoking prevalence.

This review aims to synthesize Türkiye’s comprehensive tobacco control measures, including legislation, smoking cessation initiatives, public awareness campaigns, and taxation policies. The study highlights the tobacco industry’s influence in Türkiye in terms of tobacco control.

## METHODS

This review synthesizes data from various sources, including national health surveys (Türkiye Health Surveys), global surveys (Global Adult Tobacco Survey, Global Youth Tobacco Survey), and reports from the Turkish Ministry of Health and the Global Burden of Disease. The selection focused on relevance, recency, and credibility, prioritizing data from reputable sources published within the last decade. Data extraction involved identifying, screening, and reviewing full-text articles, followed by categorizing key data points into themes such as prevalence rates and policy impacts. The review focused on the WHO FCTC and MPOWER strategy for assessing the adherence to tobacco control, as well as national activity reports.

**Table 1:** Prevalence (%) of daily tobacco smoking among adults in Türkiye according to different data sources, 1988–2019 (Sources: CDC, TurkStat, WHO, TEPAV^2,14-16,18^)

*Year*	*THDS* *^17^*	*GATS*	*STEPS*	*THS*
1988**	43.6*			
**1993**	33.6*			
2003**	33.8			
**2008**		27.4		
**2010**				25.4
**2012**		23.8		23.2
**2014**				27.3
**2016**		29.6		26.5
**2019**			29.2	28.0
**2022**				28.3

* The original source does not clarify if the stated smoking rates encompass only daily smokers or include both daily and occasional smokers.

** The data from 1988, 1993, and 2003 pertain to adults aged >18 years, whereas the statistics for other years in the table apply to adults aged >15 years.

## RESULTS

### Tobacco use profile in Türkiye

Global Burden of Disease 2019 Türkiye Report results showed that tobacco attributable disability-adjusted life years (DALY) per 100000 population increased from 3283 in 2002 to 3407 in 2019, reported by Health Statistics Yearbook 2021 by the Turkish Ministry of Health^12^.

Comprehensive adjusted statistical analysis of tobacco uses in Türkiye, produced by Türkiye Ekonomi Politikaları Araştırma Vakfı-TEPAV (Türkiye Economy Policies Research Foundation), can be found in a report named ‘The Economics of Curbing Smoking in Turkey: A Scoping Review Supply, Demand, Health, and Public Policy Aspects’ with the help of a grant by Foundation for a Smoke-Free World, Inc.^13^. The report relies on microdata from national surveys to provide a detailed understanding of consumption patterns. The surveys Türkiye Health Surveys (THS)^14^, Global Adult Tobacco Survey (GATS)^15^, Global Youth Tobacco Survey (GYTS)^16^, Türkiye Demographic and Health Surveys (TDHSs)^17^, and STEPwise approach to Surveillance (STEPS)^18^, offer insights into tobacco use by various demographics, reasons for smoking, and the impact of educational level on smoking prevalence.

The tobacco industry’s funding and other forms of relationships with the Foundation for a Smoke-Free World, Inc., and the Foundation’s Global Action to End Smoking in May 2024, as evidenced by the Tobacco Tactics initiative from the University of Bath^19^, are well documented.

The TEPAV report indicates that although there have been periods where smoking prevalence decreased, the overall trend does not show a consistent and sustained reduction in tobacco use in Türkiye. The analysis highlights several key findings, as summarized in Table 2.

**Table 2:** Key findings from the TEPAV report

*Key finding*	*Explanation*
**Gender disparities**	In 2016, 40.1% (41.3% in 2022) of adult men and 13.3% (15.5% in 2022) of adult women were daily smokers. The gap in smoking prevalence between men and women is narrowing, with a significant increase in smoking rates among women over time.
**Youth smoking**	In 2016, 12% of youth aged 15–17 years were daily smokers. There has been an increasing trend in tobacco use among youth aged 13–15 years, with rates rising from 8.4% in 2003 to 17.9% in 2017.
**Initiation age**	In 2016, 52.4% of daily smokers in Türkiye reported starting smoking before reaching the legal age of 18 years.
**Education**	The prevalence of smoking increases with education level in Türkiye. In 2016, 38.2% of university graduates were smokers, compared to 11.0% among those without formal schooling.
**Geography**	In 2016, regions like Western Marmara, Istanbul, and Eastern Marmara had high smoking rates (>35%), whereas the Western Black Sea region had the lowest rate at 19.3%.
**Type of tobacco product used**	The prevalence according to type of product were: 30.1% manufactured cigarettes, 2.3% hand-rolled cigarettes, 29.6% any other.

**Figure 2:**
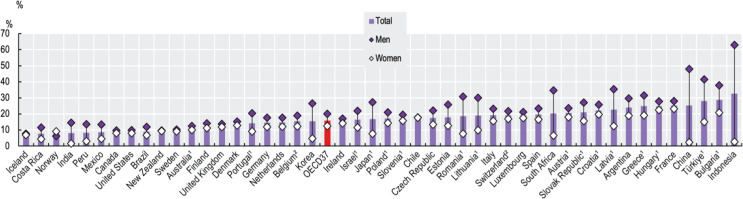
Prevalence of daily smoking for the population aged 15 years and over, by sex, 2021 (Source: OECD Health Statistics 2023^11^)

Current data make Türkiye the third country with the highest smoking rates among OECD countries (Figure 2). Vapes and other electronic cigarettes are prohibited in the country, leading to the country’s exclusion from OECD vaping statistics. The percentages of types of tobacco products being consumed by daily smokers are presented in THS (2016). The THS report also includes a category for ‘any other’ forms of tobacco use, the specifics of which are not clearly defined, as indicated in the final row of Table 2. However, other forms of tobacco are likely to refer to hookah (narghile), and electronic cigarettes, vapes that are obtained through smuggling; therefore, data on vaping and e-cigarettes remain a gap in the sources because 29.6% of daily smokers claim that they use other forms of tobacco.

### Strategies, activities, and stakeholders

Türkiye has been actively engaged in tobacco control since implementing its initial regulations in 1996, which included prohibitions on smoking in certain public areas and a mandate for television networks to broadcast 90 minutes monthly on tobacco and its dangers. In 2008, Türkiye further strengthened its tobacco control efforts by developing its first national strategy document and a five-year action plan, adopting the WHO MPOWER model. The Strategic Planning and Action Plan underwent updates in 2012 and again in 2018^20^. Türkiye’s approach to tobacco control was highlighted as a model case in the 2021 Global Progress Report of the WHO Framework Convention on Tobacco Control^21^ and received a ‘high level of compliance’ rating with the MPOWER measures in the 2021 WHO Country Profile report on the global tobacco epidemic^22^.

Following Türkiye’s adoption of the MPOWER model, tobacco control initiatives shifted focus to this comprehensive approach, moving beyond basic legislative and regulatory changes. MPOWER includes six key components: Monitoring tobacco use and prevention policies (M); Protecting people from tobacco smoke (P); Offering help to quit tobacco use (O); Warning about the dangers of tobacco (W); Enforcing bans on tobacco advertising, promotion, and sponsorship (E); and Raising taxes on tobacco products (R). This strategic framework has been central to intensifying tobacco control efforts in Türkiye.

Smoking Cessation initiatives were predominantly coordinated and run by the Turkish Ministry of Health, Directorate of Public Health^23^. Academia, several NGOs, and the WHO Country Office have participated in and supported Tobacco Control Campaigns^24^.

Tobacco Control Strategy Document and Action Plan 2018–2023 is the initial strategy developed by a range of consortia to combat tobacco use^20^. The action plan focuses on reducing the demand for tobacco products, protecting children and adolescents from tobacco use, coordinating and monitoring tobacco control efforts, and enhancing tobacco product regulation^25^. Key initiatives include raising awareness, supporting smoking cessation, increasing taxation, and implementing comprehensive smoke-free policies^26^. The plan also addresses the illegal tobacco trade, strengthens enforcement measures, and enhances coordination among various stakeholders^27^. Table 3 gives a list of activities implemented by stakeholders as part of the action plan in the 2018 Tobacco Control Report.

**Table 3:** Activities implemented by stakeholders according to the 2018 Tobacco Control Report^28^

*MPOWER*	*Activities*	*Stakeholders*
**Monitoring**	GATS, GYTS, THS, THDS, STEPS Surveys	WHO, Ministry of Health, Academia
**Protecting**	Implementing smoke-free laws in all closed public spaces, developing a robust inspection system, and launching initiatives like the Green Detector mobile app for reporting violations.	Turkish Parliament, Ministry of Health
**Offering**	Providing support for quitting tobacco through smoking cessation clinics with over 400 thousand appointments in 2018 and the ‘Hello 171’ Quit Smoking Advice Line.	Ministry of Health
**Warning**	Running media campaigns, launching websites, funding activities, and educational programs to raise awareness about the harms of tobacco.	Yeiş lay, among other NGOs, the Ministry of Health and Youth, Media Outlets, Ministry of Education
**Enforcing**	Plain package, bans on tobacco advertising, promotion, and sponsorship, and penalizing violations.	Turkish Parliament, Ministry of Health and Finance
**Raising taxes**	A tax of 82.5% on tobacco products, ban on electronic cigarettes, vapes, and flavors.	Turkish Parliament, Ministry of Health and Finance

Another important factor in smoking cessation in Türkiye is Yeşilay. The Green Crescent Society (Yeşilay) is an independent, non-profit organization in Türkiye dedicated to preventing all types of addiction. Engaging in various activities such as advocacy, rehabilitation and counseling, education and training, and volunteering, Yeşilay has a broad presence throughout Türkiye, with 105 offices in all 81 provinces. In 2022, the organization conducted training for >70000 individuals and provided smoking cessation counseling to >4000 adults^29^.

### Challenges and recommendations

Türkiye, long known for tobacco production and consumption, is experiencing a shift in public perception. Increasing awareness of tobacco’s health risks, including its impact on both users and those exposed to secondhand smoke, is leading to greater support for anti-tobacco measures and smoke-free environments^30^. Despite this growing awareness, the tobacco industry continues to find new marketing strategies to maintain its market^31^. Therefore, it is crucial for tobacco control efforts in Türkiye to be dynamic, interactive, and responsive to emerging needs, continuously adapting to combat the industry’s evolving tactics.

One significant challenge in tobacco control in Türkiye is the inconsistent and often limited impact assessments of the implemented measures. While certain evaluations have shown positive outcomes, such as reductions in smoking prevalence and heightened public awareness, comprehensive assessments of recent policies are lacking. This inconsistency hampers the ability to fully understand the effectiveness of various interventions. For example, while national health surveys and global studies provide some insights, they do not cover all aspects of tobacco control measures. The absence of systematic and comprehensive impact evaluations remains a significant obstacle, limiting the ability to refine and enhance tobacco control strategies effectively. Addressing this gap through rigorous impact assessments is crucial for identifying successful interventions and areas needing improvement, ultimately strengthening tobacco control efforts in Türkiye.

Tobacco control must be continuous and comprehensive, focusing on key priorities and undergoing regular monitoring and revision. Strengthening ongoing anti-tobacco activities is crucial for maintaining a smoke-free environment. Immediate attention is needed in areas like tax increases and smoking cessation support, while other strategies must persist without interruption^32^.

As the world’s most smuggled legal consumer product, combating the illegal tobacco trade is vital. In Türkiye, this issue affects producers, consumers, and the community. A stronger system is needed to prevent the sale of smuggled and illegal tobacco products, which attract consumers seeking cheaper options^25,32^.

The increasing prevalence of narghile (hookah) smoking among the youth in Türkiye is particularly alarming. Although national surveys did not cover other forms of tobacco, various research has documented a significant rise in hookah use among adolescents, highlighting a shift in tobacco consumption trends^33^. A pervasive and incorrect belief is that narghile smoking is safer than cigarette smoking or that it does not involve tobacco. Contrary to this misconception, recent studies have established that narghile smoking exposes users to substantial levels of harmful toxins and presents health risks comparable to those of cigarette smoking, including respiratory and cardiovascular diseases^34^. Moreover, the communal nature of narghile use, involving shared mouthpieces, exacerbates the risk of communicable diseases. These health risks underscore the urgent need for targeted public health interventions and stricter regulatory measures to mitigate the rise of narghile smoking among young people.

Over 400 smoking cessation centers exist in Türkiye, but their distribution is uneven. Integrating smoking cessation services into primary healthcare and involving family physicians in assessing patients’ smoking behaviors can improve accessibility.

Implementation of smoke-free policies needs stricter regulation across all public spaces, with improved methods for monitoring and addressing violations.

Effective control measures are required to limit the influence of the tobacco industry through advertising and sponsorship^35^.

Non-governmental tobacco control relies heavily on voluntary work, lacking specific funding. Allocating funds for these activities would bolster their effectiveness. The tobacco industry in Türkiye, like in many other countries, has been actively involved in influencing tobacco control policies, often in ways that undermine public health initiatives. Article 5.3 of the WHO Framework Convention on Tobacco Control (FCTC) mandates that policies should be protected from the commercial and vested interests of the tobacco industry. The lack of specific regulations on the tobacco industry and policy implementation gap analysis of tobacco control, may hinder the addressing of commercial determinants of health.

The industry employs several strategies to influence policy, including lobbying, funding research that downplays the risks of tobacco use (e.g. TEPAV report), and engaging in corporate social responsibility (CSR) initiatives to improve its public image. These tactics aim to create a favorable regulatory environment, delay the implementation of stricter controls, and maintain its market presence. For example, tobacco companies have been known to lobby against tax increases and vape bans through well-known journalists, and commentators arguing that such measures would have adverse economic impacts in the hyperinflation economic setting^36^.

Positioning Türkiye’s tobacco control efforts within a global context highlights unique challenges and strategies that might offer insights for other countries. A significant originality in the Turkish setting is the pervasive issue of tobacco smuggling, which undermines the effectiveness of tobacco control policies. Similarly, despite comprehensive legislative frameworks, including strict bans on smoking in public places and significant tax increases on tobacco products, the enforcement of these regulations is often inconsistent. Policy implementation gaps and impact assessment of such measures also remain as a limitation.

### Limitations

Limitations of this review include data gaps and potential biases from industry-funded reports.

## CONCLUSIONS

Türkiye’s journey in tobacco control represents a significant effort to combat the public health crisis posed by tobacco use. Through the implementation of WHO FCTC guidelines and MPOWER strategies, Türkiye has achieved notable progress in reducing tobacco consumption, addressing gender disparities, and tackling youth smoking. Despite these achievements, challenges such as tobacco smuggling, the popularity of narghile smoking, and the need for more accessible smoking cessation services, persist. Moving forward, Türkiye must continue to strengthen its tobacco control measures, ensuring comprehensive strategies and robust enforcement to further reduce tobacco use and its associated health and economic burdens.

## Data Availability

The data supporting this research are available from the author on reasonable request.
